# Organophosphate Pesticide Exposure and Semen Quality in Healthy Young Men: A Pilot Study

**DOI:** 10.3390/antiox14101158

**Published:** 2025-09-24

**Authors:** Jenisha L. Stapleton, Sarah Adelman, Bobby B. Najari, Kurunthachalam Kannan, Vittorio Albergamo, Linda G. Kahn

**Affiliations:** 1 Department of Population Health, NYU Grossman School of Medicine, New York, NY 10016, USA; jenisha.stapleton@nyulangone.org; 2 Department of Pediatrics, NYU Grossman School of Medicine, New York, NY 10016, USA; 3 Department of Urology, NYU Grossman School of Medicine, New York, NY 10016, USA; 4 Department of Environmental Health Sciences, College of Integrated Health Sciences, University at Albany, Albany, NY 12222, USA; 5 Department of Pediatrics, Division of Environmental Pediatrics, NYU Grossman School of Medicine, New York, NY 10016, USA; vittorio.albergamo@nyulangone.org; 6 Departments of Pediatrics and Population Health, NYU Grossman School of Medicine, New York, NY 10016, USA

**Keywords:** organophosphate pesticides, semen quality, oxidative stress, sperm concentration, oxidation-reduction potential

## Abstract

This cross-sectional pilot study aimed to examine associations between urinary metabolites of organophosphate (OP) pesticides and semen quality in 42 healthy young men. Participants answered questionnaires, provided semen and urine samples, and had anthropometric measures taken. Urine and seminal plasma were assayed for dialkylphosphate (DAP) metabolites of OP pesticides using high-performance liquid chromatography-electrospray ionization-tandem mass spectrometry. Semen quality parameters were analyzed according to the World Health Organization protocol, and seminal oxidative stress was assayed using MiOXSYS, a galvanic cell-based technology that yields an integrated measure of oxidants and antioxidants. Associations of OP pesticide metabolites with continuous and dichotomous sperm concentration, percent motility, and percent normal morphology, and with seminal oxidative-reduction potential (ORP) were analyzed statistically. OP pesticide exposure was associated with lower overall semen quality. Specifically, ∑DAP metabolites, driven by diethyl metabolites, was inversely associated with percent sperm motility, but this relationship was not mediated by seminal ORP. Seminal ORP was inversely associated with sperm concentration, but OP pesticide exposure was not associated with seminal ORP.

## 1. Introduction

Approximately half of all cases of reported infertility in the United States involve male-factor infertility [1], which is commonly diagnosed via standardized analysis of measures of semen quality that include sperm concentration, motility, and morphology [2]. Poor semen quality has been associated with chronic diseases, morbidity, and overall mortality [3,4,5,6,7]. Evidence suggests a temporal decline in semen quality over the past century, especially in industrialized nations [8]. This trend coincides with an increase in industrial production of endocrine-disrupting chemicals (EDCs), many of which have been associated with lower semen quality [9]. Potential mechanisms include alterations of prostate cell physiology, including translocation of steroid hormone receptors, dysregulation of mitochondrial function, and perturbance of redox homeostasis, as demonstrated in experimental studies of exposure to dibutylphthalate and glyphosate [10,11]. In this pilot study, we investigated relations between organophosphate (OP) pesticides and semen quality in an unselected population of young men with non-occupational exposure, hypothesizing negative associations mediated by seminal oxidative stress.

Although best known as neurotoxins, OP pesticides also have endocrine-disrupting properties. Banned for residential pest management in the United States in 2000, OP pesticides are still widely used in agriculture, and the general population is mostly exposed through diet. OP pesticides have been linked not only to lower sperm concentration, but also to lower percent motility and percent normal morphological forms, parameters that are considered better predictors of male fertility than concentration [12]. Most studies of OP pesticides and semen quality have been conducted among men who were occupationally or residentially exposed and have consistently shown associations with lower semen quality [13,14,15,16,17]. These studies have generally relied on self-reported pesticide use or occupational status to determine exposure. The few studies conducted among highly exposed populations have yielded similar results [18,19,20]. The only studies to examine associations between semen quality and biomarkers of exposure to OP pesticides at levels found in the general population have been conducted among men recruited from infertility clinics. These, too, have shown associations with higher odds of poor semen quality [21,22], suggesting that even at legally permissible environmental levels, OP pesticide exposure may affect semen quality. To date there have been no biomarker studies among healthy men from the general population to test this hypothesis.

OP pesticide exposure has been linked to oxidative stress across both epidemiologic and experimental literature. One study found that oxidative stress in the blood of farm workers following intensive pesticide exposure resulted in stimulated antioxidant enzymes, indicating a response to elevated oxidative stress [23]. In another study that included Czech adults and children from the general population, urinary 3,5,6-trichloro-2-pyridinol (TCPY, a metabolite of the OP pesticide chlorpyrifos) was positively associated with oxidative stress biomarkers [24]. Biological mechanisms that could explain observed associations between OP pesticides and lower semen quality include lipid peroxidation, which not only increases reactive oxygen species (ROS) [25,26] but also compromises sperm membranes, negatively affecting both motility and morphology [27]. In addition, ROS activate at least five independent signaling pathways [28], including apoptosis of mitochondria, which are essential for sperm motility.

In summary, experimental studies have demonstrated that OP pesticide exposure negatively affects the male reproductive system [29] and human occupational studies have shown that high-dose exposure is associated with lower semen quality [30]. However, to date it has not been examined whether exposure at routine levels among healthy men from the general population affects semen quality. This pilot project was the first study to evaluate associations between concentrations of dialkylphosphate (DAP) metabolites, non-specific urinary biomarkers of OP pesticide exposure, and semen parameters among men from the general population. We hypothesized that OP pesticide metabolites would be negatively associated with sperm concentration, motility, and normal morphology, and that these associations would be mediated by elevated seminal oxidative stress, which is considered an important mechanism underlying idiopathic male-factor fertility [31,32].

## 2. Materials and Methods

### 2.1. Participant Recruitment and Study Design

Potential study participants were recruited via flyers and word of mouth. Eligibility criteria included male sex, ability to communicate in English, age 18–45, and willingness to follow the study protocol. Those with a history of relevant medical or urological conditions (e.g., urinary tract infections, varicocele, erectile dysfunction) or use of medications affecting fertility (e.g., spironolactone, ketoconazole, methotrexate) were excluded from participation.

If the prospective participant was eligible and interested, he was consented into the study and emailed a link to the baseline survey, which included questions about demographics, health history, lifestyle and behaviors, substance use, physical activity, sleep quality, stress, anxiety, and depression. After the enrolled participant completed the baseline questionnaire, study personnel called to schedule two same-day appointments for data collection: a visit to a local commercial andrology lab for provision of a semen sample (Repro Lab, Inc., New York, NY, USA) and a clinic visit where study staff conducted anthropometric measures and obtained a urine sample, which was aliquoted and stored at −80 °C.

Participants were compensated for their time and a snowball sampling technique was used that incentivized enrolled participants to recruit acquaintances to join the study. Data were collected from 45 study participants between September 2022 and January 2023. This study was approved by the Institutional Review Board (IRB) of the New York University Grossman School of Medicine.

### 2.2. Semen Collection and Assessment of Semen Quality

Following two to seven days of ejaculatory abstinence, each participant provided a fresh semen sample that was collected via masturbation into a sterile wide-mouthed specimen cup. After liquefaction at room temperature, semen parameters, including sperm concentration (10^6^ sperm/mL), motility (percent motile sperm), and morphology (percent normal morphology according to strict criteria), were evaluated according to World Health Organization (WHO) guidelines [33]. Leftover semen was transported to New York University Langone Health (NYULH) and stored at −80 °C.

### 2.3. Measurement of Seminal Oxidation-Reduction Potential (ORP)

Frozen semen samples were thawed at room temperature then analyzed for seminal oxidation-reduction potential (ORP) using the Male Infertility Oxidative System (MiOXSYS; Caerus Biotech, Vilnius, Lithuania). ORP, measured in millivolts (mV), is an integrated measure of the existing balance between total oxidants and reductants in a biological system. To obtain this measurement, 30 μL of semen is applied to a disposable platinum-based sensor that is inserted into the MiOXSYS analyzer, where it completes an electrochemical circuit when it fills the Ag/AgCl reference cell. Higher ORP indicates higher oxidants relative to antioxidants, hence higher total oxidative stress [34]. Each sample was measured twice, and the average ORP value was normalized to sperm concentration to control for differences in cell numbers, yielding a measure in mV/10^6^ sperm/mL.

### 2.4. Measurement of Urinary Dialkylphosphate (DAP) Metabolites

DAP metabolites are non-specific metabolites of OP pesticides. Urine samples were assayed for concentrations of six DAP metabolites, the sum of which may be considered a proxy measure of total OP pesticide exposure [35,36,37]. These included three dimethyl (DM) metabolites (dimethylphosphate [DMP], dimethylthiophosphate [DMTP], and dimethyldithiophosphate [DMDTP]) and three diethyl (DE) metabolites (diethylphosphate [DEP], diethylthiophosphate [DETP], and diethyldithiophosphate [DEDTP]).

A 1.0 mL aliquot of urine was spiked with labelled internal standards and extracted using solid phase extraction. Analytes were identified and quantified using high-performance liquid chromatography-electrospray ionization-tandem mass spectrometry (HPLC-ESI-MS/MS). Quantification was achieved through an isotope dilution method. Urinary creatinine was also determined using HPLC-MS/MS. The laboratory participated in several quality assurance and quality control schemes to validate the methods [36,38].

The limits of detection (LODs) of DMP, DMTP, DMDTP, DEP, DETP, and DEDTP were 0.026, 0.013, 0.039, 0.029, 0.020, and 0.039 ng/mL, respectively. More than 50% of the samples had DMDTP and DEDTP concentrations below the LOD, so these metabolites were excluded from further analysis. The values <LOD for the remaining metabolites were imputed as LOD√2. The molar concentration of each metabolite was calculated using the formula:

((concentration in ng/mL) × (1/molecular weight) × (10^3^)).

The molar concentrations of DMP and DMTP were then summed to yield the total molar concentration of DM metabolites (∑DM) and the molar concentrations of DEP and DETP were summed to yield the total molar concentration of DE metabolites (∑DE). The sum of ∑DM and ∑DE yielded the total urinary DAP metabolite molar concentration (∑DAP).

Adjustment for urine dilution was performed via the Boeniger et al. method using creatinine [39]. Specifically, the chemical concentrations were multiplied by the ratio of the batch-specific median creatinine concentration for the study sample to the observed creatinine concentration.

### 2.5. Statistical Analysis

Sperm concentration and ∑DAP, ∑DM, and ∑DE metabolite concentrations were transformed using natural logarithms to normalize their distributions to meet model specifications and reduce the influence of outliers. ORP was dichotomized as normal (≤1.76 mV/10^6^ sperm/mL) vs. high (>1.76 mV/10^6^ sperm/mL). This threshold represented the 75th percentile of the seminal ORP distribution for this group of men. Semen parameters were dichotomized as low vs. normal according to WHO 1999 reference values, which are clinically predictive of subfecundity: sperm concentration < 20 × 10^6^/mL, percent motile sperm < 50%, and percent normal morphology < 30% [2]. We also created a dichotomous variable for overall semen quality that indicated participants who had low categorization for any one of the three parameters (concentration, motility, or morphology).

Potential covariates included variables associated with semen quality in prior literature: race-ethnicity (a proxy for socioeconomic status as related to structural racism in the United States), age, current marijuana use, current alcohol use, current nicotine use, underwear type, sleep duration, perceived stress level, depression/anxiety, and body mass index (BMI). BMI (kg/m^2^) was calculated from anthropometric measures and categorized as underweight (BMI < 18.5), normal (BMI ≥ 18.5 and BMI < 25), overweight (BMI ≥ 25 and BMI < 30), and obese (BMI ≥ 30). One participant was underweight (BMI = 17.6) and two were extremely/class 3 obese (BMI = 42.9, 44.0). Because low BMI and high BMI are strongly correlated with male subfecundity [5,6], we were concerned that these three participants would skew the results of our analysis due to the small sample size of our study, so we excluded them from further analysis.

We conducted bivariate analyses examining associations between potential covariates and both the dichotomous ORP and overall semen quality variables using independent sample *t*-tests for continuous variables and Fisher’s exact test for categorical variables. Linear and logistic regressions were used to estimate associations of DAP metabolite concentrations with continuous and dichotomous measures of ORP and semen quality, respectively. All analyses were conducted using R version 4.4.2 [40].

## 3. Results

Baseline characteristics of the 42 men included in the statistical analyses are shown in Table 1. The majority were non-Hispanic White (78%), and the mean (SD) age was 29.5 (3.79) years. The median [minimum, maximum] BMI was 24.1 [19.5, 39.2]; 61.9% of the men had a normal BMI, 28.6% were overweight, and 9.5% were obese. Ten (24%) had high ORP (ORP > 1.76 mV/10^6^ sperm/mL) and 32 (76%) had normal ORP (ORP ≤ 1.76 mV/10^6^ sperm/mL). There were no significant differences among these or other potential covariates, such as underwear type, sleep duration, substance use, perceived stress, and depression/anxiety, between those with high ORP and normal ORP. As such, subsequent analyses were not adjusted for any covariates. Table 1 also presents the distributions of semen quality parameters and ORP in the overall sample and among those with high and normal ORP. The proportion of men with normal sperm concentration was significantly lower in the high ORP group compared with the normal ORP group (continuous: *p* < 0.001, categorical: *p* = 0.04). Other parameters (motility, morphology, and overall semen quality) did not differ significantly between the normal and high ORP groups.

Detection rates and distributions of urinary DAP metabolite concentrations are presented in Table 2. Among the urine samples of the 42 participants, 100% of DEDTP and 88.9% of DMDTP concentrations were <LOD, hence they were excluded from further analysis. The median urinary concentration for DEP was 2.45 ng/mL. This measurement falls within the 95% confidence interval of the 50th percentile, 1.94–2.47 ng/mL, for adult men in the United States from the 2017–2018 National Health and Nutrition Examination Survey (NHANES) [41]. The median concentration for DMP was 0.67 ng/mL. This measurement is half of the DMP metabolite concentration, 1.34 ng/mL, observed in the 2017–2018 NHANES, perhaps reflecting the reduction in use of DM OP pesticides, such as dichlorvos and malathion, in recent years.

**Figure 1:**
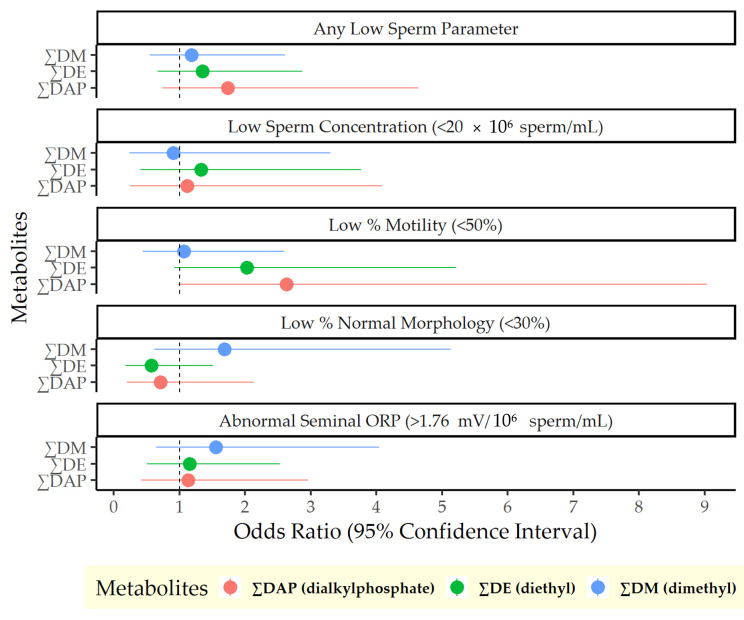
Odds of poor semen quality per log-unit increase in urinary dialkylphosphate metabolite concentrations.

Results from the logistic regression analyses of OP pesticide exposure and semen quality are presented in Figure 1. A 1 log-unit increase in ∑DAP was associated with a 74% higher odds of any low semen parameter (odds ratio [OR]: 1.74, 95% confidence interval [CI]: 0.74, 4.64), driven by the DAP metabolites’ association with motility: A 1 log-unit increase in ∑DAP was associated with 163% higher odds of low percent motile sperm (OR: 2.63, 95% CI: 0.98, 9.03). This finding was entirely due to DE metabolites, which were likewise associated with higher odds of low percent motile sperm (OR: 2.03, 95% CI: 0.92, 5.21). Results from the linear regression models were consistent in direction, although not statistically significant. In all cases, confidence intervals were wide due to the small sample size. Complete results of both linear and logistic models are included in the Supplementary Materials.

Unadjusted linear and logistic associations of continuous seminal ORP with continuous and dichotomized semen parameters, respectively, are presented in Table 3. Seminal ORP was negatively associated with sperm concentration. For every 1 log-unit increase in seminal ORP, sperm concentration decreased by 0.48 × 10^6^ sperm/mL (95% CI: −0.62, −0.33). Similarly, a 1 log-unit increase in seminal ORP was associated with 3.95 times the odds of low sperm concentration (95% CI: 1.41, 18.93).

## 4. Discussion

This cross-sectional pilot study is not only the first ever to investigate associations of OP pesticides with semen quality among young men from the general population but also tests a novel measure of oxidative stress as a potential mediator. We hypothesized that OP pesticide exposure would be associated with lower semen quality and that the association would be mediated by increased oxidative stress, operationalized as seminal oxidation-reductive potential (ORP), an overall measure of oxidative stress that has been linked to semen quality and fertility outcomes [42]. We measured ORP using MiOXSYS [43], an inexpensive and comprehensive system for quantifying ORP in human semen [44]. To estimate OP pesticide exposure, we measured urinary DAP metabolites.

In this study, OP pesticide exposure was associated with lower semen quality, driven by an inverse association between ∑DAP metabolites and normal sperm motility. This relationship was not mediated by seminal ORP, however, which was not associated with OP pesticide exposure or sperm motility. Seminal ORP was inversely associated with sperm concentration, modeled both continuously and dichotomously, in keeping with findings from prior studies [45,46,47].

Our main finding, that routine OP pesticide exposure was related to lower sperm motility, is particularly relevant to population health because among conventional semen parameters, motility is considered by many to be the most closely linked with male fecundity [48]. There are many potential biological mechanisms that could explain this association. First, OP pesticides are effective insecticides because they inhibit acetylcholinesterase, resulting in excessive accumulation of the neurotransmitter acetylcholine (ACh) [49]. The cholinergic signaling system plays an important role in sperm motility, as ACh binds to nicotinic ACh receptors in sperm flagella that mediate calcium channel signaling, increasing intracellular calcium and inducing sperm motility [50]. Excess intracellular calcium has been shown to cause mitochondrial failure and reduced motility in experimental studies [51,52]. Second, OP pesticides have been shown to reduce sperm mitochondrial membrane potential [53], resulting in reduced energy production, increased oxidative stress, and impaired motility. Third, OP pesticides interrupt the normal assembling of tail structural protein components necessary for motility [54]. Finally, OP pesticides induce lipid peroxidation [55], which has been shown to be higher in the seminal plasma of men with lower sperm motility [56].

Although we did not detect an association between oxidative stress and motility, so cannot conclude that it mediates this relationship, we observed an association between ORP and lower sperm concentration that, again, can be attributed to a variety of potential pathways. Excess ROS can damage sperm DNA, lipids, and proteins that in turn impair spermatogenesis [57,58]. ROS also induce apoptosis of spermatozoa. Epidemiologic studies have demonstrated a higher percentage of apoptotic sperm in men who have low sperm concentration compared with men who have normal sperm concentration [59]. Furthermore, results from a meta-analysis demonstrated a consistent association between oxidative stress biomarkers and low sperm concentration across diverse populations [60].

This study has some limitations. Due to the small sample size, most observed associations did not reach statistical significance. Also, the seminal ORP threshold used in this study, 1.76 mV/10^6^ sperm/mL, was based on the third quartile of the seminal ORP distribution for this group of men, a common, but crude statistical approach for determining critical values. It is possible that this threshold may not be the optimal threshold for determining semen quality in healthy men. Nguyen et al. found that the optimal ORP threshold for determining semen quality was 0.77 mV/10^6^ sperm/mL using receiver operating characteristic curves for infertile men in Vietnam [47], while 1.34 mV/10^6^ sperm/mL has been regarded as a clinical cutoff value for categorizing normal and abnormal ORP groups based on a study of more than 2000 patients seeking fertility assistance [34]. To our knowledge, there is not an established critical value for ORP in healthy young men, so we used a data-driven method instead. Finally, DAP metabolites of OP pesticides are non-specific; hence it is not possible to identify individual compounds associated with lower semen quality in this study.

A major strength of this study is that it is the first to analyze associations between OP pesticide exposure and semen quality in healthy young men from the general population and not infertile men. The only prior study of routine pesticide exposure and semen quality among healthy men, which used self-reported diet to assess overall pesticide exposure, found that men with greater intake of fruit and vegetables in the low-to-moderate pesticide residue category had higher sperm concentration and total sperm count than men with lesser intake [61]. This could have been due to the beneficial effects of fruits and vegetables, which may outweigh the adverse effect of the pesticides. Another strength of this study is that the exposure assessment was based on biospecimen data and not self-report or occupational exposure. Additionally, seminal oxidative stress was measured using a novel measure that accounts for the presence of both oxidants and antioxidants in the target tissue.

## 5. Conclusions

This study contributes to the literature by examining the extent to which routine exposure to OP pesticides, common in the conventional food supply [62], may be negatively associated with semen quality in healthy men from the general population. Specifically, we found OP pesticide exposure to be inversely associated with sperm motility, which many consider to be the parameter most predictive of successful fertilization [48]. Future studies are warranted to replicate these findings in larger samples and build an evidence base for more stringent regulation of OP pesticides based on their potential for reproductive toxicity.

## Figures and Tables

**Table 1:** Baseline characteristics and semen quality parameters by seminal oxidation-reduction potential (ORP).

Characteristic	Overall (N = 42) *	High ORP >1.76 mV/10^6^ Sperm/mL (N = 10)	Normal ORP ≤1.76 mV/10^6^ Sperm/mL (N = 32) *	*p*-Value ^+^
Race-Ethnicity **				
Hispanic	4 (9.8%)	0 (0%)	4 (12.9%)	0.81
Other race, NH	5 (12.2%)	1 (10.0%)	4 (12.9%)	
White, NH	32 (78.0%)	9 (90.0%)	23 (74.2%)	
Current Marijuana Use				
No	18 (43.9%)	3 (30.0%)	15 (48.4%)	0.47
Yes	23 (56.1%)	7 (70.0%)	16 (51.6%)	
Current Alcohol Use				
No	16 (39.0%)	4 (40.0%)	12 (38.7%)	1
Yes	25 (61.0%)	6 (60.0%)	19 (61.3%)	
Current Nicotine Use				
No	23 (56.1%)	5 (50.0%)	18 (56.3%)	0.72
Yes	18 (43.9%)	5 (50.0%)	13 (40.6%)	
Underwear Type				
Combination of loose and tight-fitting underwear	11 (26.8%)	5 (50.0%)	6 (19.4%)	0.14
Briefs or tight-fitting underwear	24 (58.5%)	4 (40.0%)	20 (64.5%)	
Loose boxer shorts or No underwear	6 (14.6%)	1 (10.0%)	5 (16.1%)	
Sleep Duration (hours)				
Less than 6	6 (14.6%)	0 (0%)	6 (19.4%)	0.18
6–7	12 (29.3%)	2 (20.0%)	10 (32.3%)	
7 or more	23 (56.1%)	8 (80.0%)	15 (48.4%)	
Perceived Stress Level				
Low	13 (31.7%)	4 (40.0%)	9 (28.1%)	0.70
Moderate	28 (68.3%)	6 (60.0%)	22 (68.8%)	
Age (years)				
Mean (SD)	29.5 (3.79)	30.1 (4.18)	29.3 (3.71)	0.61
Median [Min, Max]	29.6 [23.5, 36.3]	30.2 [23.5, 36.3]	29.2 [23.6, 36.3]	
Body Mass Index (BMI, kg/m^2^)				
Mean (SD)	24.9 (3.61)	24.2 (1.51)	25.2 (4.03)	0.24
Median [Min, Max]	24.1 [19.5, 39.2]	23.8 [21.9, 27.3]	24.2 [19.5, 39.2]	
BMI Categories				
Normal	26 (61.9%)	8 (80.0%)	18 (56.3%)	0.48
Overweight	12 (28.6%)	2 (20.0%)	10 (31.3%)	
Obese	4 (9.5%)	0 (0%)	4 (12.5%)	
Depression/Anxiety (GSI score)				
Mean (SD)	30.6 (9.93)	28.9 (8.54)	31.1 (10.4)	0.51
Median [Min, Max]	29.0 [18.0, 63.0]	27.5 [18.0, 45.0]	29.0 [18.0, 63.0]	
Creatine-adjusted ∑DAP, nmol/mL				
Mean (SD)	56.2 (75.2)	45.5 (19.1)	59.6 (85.6)	0.39
Median [Min, Max]	40.4 [8.66, 492]	39.2 [20.2, 80.0]	40.9 [8.66, 492]	
Sperm Concentration (10^6^/mL)				
Mean (SD)	76.9 (52.6)	27.7 (14.3)	92.3 (50.7)	<0.001
Median [Min, Max]	61.0 [4.00, 208]	26.0 [4.00, 44.0]	83.5 [18.0, 208]	
Low (<20 × 10^6^/mL)	4 (9.5%)	3 (30.0%)	1 (3.1%)	0.04
Sperm Motility (%)				
Mean (SD)	59.0 (12.6)	58.2 (16.0)	59.2 (11.6)	0.86
Median [Min, Max]	60.5 [35.0, 81.0]	58.5 [35.0, 81.0]	60.5 [38.0, 79.0]	
Low (<50%)	10 (23.8%)	3 (30.0%)	7 (21.9%)	0.68
Morphology (%)				
Mean (SD)	45.19 (13.9)	42.2 (11.9)	46.1 (14.6)	0.40
Median [Min, Max]	45.0 [18.0, 75.0]	43.5 [22.0, 65.0]	45.5 [18.0, 75.0]	
Low (<30%)	7 (16.7%)	2 (20.0%)	5 (15.6%)	1
Overall Semen Quality				
Low ***	16 (38.1%)	5 (50.0%)	11 (34.4%)	0.47

Abbreviations: NH: Non-Hispanic; GSI: Global Severity Index for depression/anxiety; DAP: Dialkylphosphate metabolites; ORP: oxidation–reduction potential (mV/106 sperm/mL). ^+^ Continuous parameters were compared using independent sample *t*-tests. Categorical parameters were compared using Fisher’s exact test. * Questionnaire data are missing for one participant, therefore N = 41 for the overall sample and N = 31 for those with normal ORP for those variables. ** Race and ethnicity are conventional proxies for environmental exposures, socio-economic status, and structural racism, all of which are drivers of health disparities in the United States. *** Low categorization for at least one of the three parameters (concentration, motility, or morphology).

**Table 2:** Detection rates, medians, 25th and 75th percentiles of urinary DAP metabolite concentrations (N = 42).

Chemicals ^a^	% <LOD	25th Percentile	Median (50% Percentile)	75th Percentile	50% Percentile (95% CI) NHANES 2017–2018 Adult Males
DEDTP	100.0	<LOD	<LOD	<LOD	<LOD
DEP	0.00	1.33	2.45	4.62	2.18 (1.94–2.47)
DETP	46.70	<LOD	0.38	0.67	0.13 (0.10–0.16)
∑DE	-	10.87	17.26	34.18	-
DMDTP	88.90	<LOD	<LOD	<LOD	0.12 (<LOD–0.14)
DMP	0.00	0.96	0.67	1.85	1.34 (1.13–1.54)
DMTP	4.40	0.24	0.11	0.82	0.67 (0.57–0.86)
∑DM	-	6.17	9.72	25.76	-
∑DAP	-	19.21	30.90	83.73	-

Abbreviations: DEDTP: diethyldithiophosphate; DEP: diethylphosphate; DETP: diethylthiophosphate; ∑DE: total molar concentration of diethyl (DE) metabolites; DMDTP: dimethyldithiophosphate; DMP: dimethylphosphate; DMTP: dimethylthiophosphate; ∑DM: total molar concentrations of dimethyl (DM) metabolites; ∑DAP: total molar dialkylphosphate (DAP) metabolites; NHANES: National Health and Nutrition Examination Survey; % <LOD: percent below the limit of detection. The limits of detection in this study ranged from 0.01 to 0.03 ng/mL depending on the chemical, while NHANES was 0.10 ng/mL for the listed chemicals. ^a^ Units of DEDTP, DEP, DETP, DMDTP, DMP, DMTP are ng/mL. ∑DE is the molar sum of DEP and DETP. ∑DM is the molar sum DMP and DMTP. ∑DAP is the molar sum of ∑DE and ∑DM. Units of ∑DM, ∑DE, and ∑DAP are nmol/mL.

**Table 3:** Associations of seminal oxidation-reduction potential (ORP) with semen parameters.

	Sperm Concentration * (10^6^/mL)	**Motile Sperm (%)**	**Normal Morphology (%)**	
**Predictor**	beta (95% CI)	beta (95% CI)	beta (95% CI)	
Seminal fluid ORP * (mV/10^6^ sperm/mL)	−0.48 (−0.62, −0.33)	1.40 (−1.80, 4.60)	0.31 (−3.27, 3.89)	
	**Low Sperm** **Concentration** **(<20 × 10^6^ Sperm/mL)**	**Low % Motile Sperm (<50%)**	**Low % ** **Normal** **Morphology (<30%)**	**Any Low** **Parameter**
Predictor	Odds Ratio (95% CI)	Odds Ratio (95% CI)	Odds Ratio (95% CI)	Odds Ratio (95% CI)
Seminal fluid ORP * (mV/10^6^ sperm/mL)	3.95 (1.41, 18.93)	0.78 (0.42, 1.40)	1.14 (0.58, 2.26)	0.94 (0.56, 1.57)

* natural-log transformed.

## Data Availability

Deidentified data supporting the conclusions of this article will be made available by the authors on request.

## Supplementary Materials

The following supporting information can be downloaded at: https://www.mdpi.com/article/10.3390/antiox14101158/s1, Table S1: Logistic Regression Results of OP Pesticide Exposure and Semen Quality; Table S2: Linear Regression Results of OP Pesticide Exposure and Semen Quality.

## Abbreviations

The following abbreviations are used in this manuscript:

ACh	Acetylcholine
BMI	Body mass index
CI	Confidence interval
DEP	Diethylphosphate
DETP	Diethylthiophosphate
∑DE	Total molar concentration of diethyl ether (DE) metabolites
DMDTP	Dimethyldithiophosphate
DMP	Dimethylphosphate
DMTP	Dimethylthiophosphate
∑DM	Total molar concentrations of dimethyl ether (DM) metabolites
∑DAP	Total molar concentration of dialkylphosphate (DAP) metabolites
LOD	Limit of detection
MiOXSYS	Male Infertility Oxidative System
NHANES	National Health and Nutrition Examination Survey
NH	Non-Hispanic
GSI	Global severity index
OP	Organophosphate pesticide
ORP	Oxidation-reduction potential
