# Supplementary material for: Organophosphate Pesticide Exposure and Semen Quality in Healthy Young Men: A Pilot Study
Source: Antioxidants (Basel). 2025 Sep 24;14(10):1158. doi: 10.3390/antiox14101158 (PMC12562182; doi:10.3390/antiox14101158)
Supplement: Supplementary file 1 [file antioxidants-14-01158-s001.zip › antioxidants-3795012-supplementary.pdf]

Supplement

Table S1. Logistic Regression Results of OP Pesticide Exposure and Semen Quality

Metabolite	Odds Ratio	CI Lower	CI Upper	P-Value	Outcome
∑DM	0.91	0.24	3.30	0.88	Low Sperm Concentration (<20 x 10^6 sperm/mL)
∑DE	1.33	0.41	3.77	0.60	Low Sperm Concentration (<20 x 10^6 sperm/mL)
∑DAP	1.11	0.25	4.08	0.88	Low Sperm Concentration (<20 x 10^6 sperm/mL)
∑DM	1.06	0.44	2.60	0.89	Low % Motility (<50%)
∑DE	2.03	0.92	5.21	0.10	Low % Motility (<50%)
∑DAP	2.63	0.98	9.03	0.08	Low % Motility (<50%)
∑DM	1.68	0.62	5.13	0.32	Low % Normal Morphology (<30%)
∑DE	0.57	0.18	1.51	0.31	Low % Normal Morphology (<30%)
∑DAP	0.71	0.20	2.13	0.56	Low % Normal Morphology (<30%)
∑DM	1.18	0.55	2.61	0.66	Any Low Sperm Parameter
∑DE	1.35	0.67	2.87	0.41	Any Low Sperm Parameter
∑DAP	1.74	0.74	4.64	0.22	Any Low Sperm Parameter
∑DM	1.56	0.65	4.04	0.33	Abnormal Seminal ORP (>1.76 mV/10^6 sperm/mL)
∑DE	1.15	0.51	2.53	0.72	Abnormal Seminal ORP (>1.76 mV/10^6 sperm/mL)
∑DAP	1.13	0.42	2.96	0.80	Abnormal Seminal ORP (>1.76 mV/10^6 sperm/mL)

Abbreviations:
∑DE: total molar concentration of diethyl ether (DE) metabolites
∑DM: total molar concentrations of dimethyl ether (DM) metabolites
∑DAP: total molar dialkylphosphate (DAP) metabolites
CI: 95% confidence interval

Table S2. Linear Regression Results of OP Pesticide Exposure and Semen Quality

Metabolite	beta	CI Lower	CI Upper	P-Value	Outcome
∑DM	-0.05	-0.36	0.27	0.78	Sperm Concentration (10^6 sperm/mL)
∑DE	-0.13	-0.42	0.16	0.36	Sperm Concentration (10^6 sperm/mL)
∑DAP	-0.16	-0.50	0.19	0.37	Sperm Concentration (10^6 sperm/mL)
∑DM	-2.49	-7.28	2.29	0.30	% Motility
∑DE	-2.01	-6.39	2.37	0.36	% Motility

∑DAP	-3.67	-8.90	1.55	0.16	% Motility
∑DM	0.04	-5.35	5.43	0.99	% Normal Morphology
∑DE	0.58	-4.34	5.49	0.81	% Normal Morphology
∑DAP	-0.19	-6.14	5.75	0.95	% Normal Morphology
∑DM	0.19	-0.29	0.67	0.43	Seminal ORP (mV/10^6 sperm/mL)
∑DE	0.28	-0.15	0.71	0.19	Seminal ORP (mV/10^6 sperm/mL)
∑DAP	0.28	-0.24	0.80	0.283	Seminal ORP (mV/10^6 sperm/mL)

Abbreviations:

∑DE: total molar concentration of diethyl ether (DE) metabolites

∑DM: total molar concentrations of dimethyl ether (DM) metabolites

∑DAP: total molar dialkylphosphate (DAP) metabolites

CI: 95% confidence interval
